# New Phthalate Link?: DEHP Metabolites and Altered Thyroid Hormone Levels in Men

**Published:** 2007-07

**Authors:** Rebecca Renner

Human studies have shown widespread exposure to phthalates, compounds used in the manufacture of household, consumer, and medical products. The plasticizer DEHP is one of the most widely used chemicals in this class. A limited number of rat studies have linked DEHP exposure to alterations in thyroid signaling and lower plasma thyroxine (T_4_) concentrations. Now a study of adult men for the first time shows an association between higher urinary levels of the metabolite MEHP and reduced thyroid hormone in blood serum **[*EHP* 115:1029–1034; Meeker et al.]**.

Phthalates are metabolized and excreted quickly; these metabolites, rather than the parent diesters, are believed to be the active toxicants. Ingested DEHP is initially hydrolyzed in the intestine to MEHP. The metabolites MEOHP and MEHHP are then produced by the oxidation of MEHP.

The study participants included 408 men between the ages of 18 and 55. All were partners in subfertile couples who visited a Boston fertility center between January 2000 and May 2004. Each man completed a questionnaire and provided urine and blood samples on the same day. Blood samples were analyzed for free T_4_, total tri-iodothyronine (T_3_), and thyroid-stimulating hormone. Urine analysis provided data on concentrations of DEHP metabolites.

MEHP was detected in 83% of the 408 samples. MEOHP and MEHHP were found in more than 95% of 208 samples tested (the sample size was smaller because methods for analyzing these metabolites became available only later in the study).

Multivariate regression analysis revealed a statistically significant inverse association between urine MEHP concentrations and serum total T_3_ levels. In an effort to determine whether individual differences in the ability to further metabolize and neutralize MEHP might explain this relationship, the researchers also calculated the percentage of MEHP relative to the other metabolites. They found a weaker though still statistically significant inverse association between the percentage of MEHP relative to the other metabolites and free T_4_ levels.

This suggests that an individual’s ability to metabolize and neutralize MEHP may play a role in determining effects. The researchers urge other scientists to consider whether MEHP levels relative to other metabolite concentrations might serve as potential markers for metabolic vulnerability to adverse effects from DEHP exposure.

